# Changes in attitudes to awareness of hypoglycaemia during a hypoglycaemia awareness restoration programme are associated with avoidance of further severe hypoglycaemia episodes within 24 months: the A2A in HypoCOMPaSS study

**DOI:** 10.1007/s00125-022-05847-7

**Published:** 2022-12-20

**Authors:** Eduardo Sepúlveda, Peter Jacob, Rui Poínhos, Davide Carvalho, Selene G. Vicente, Emma L. Smith, James A. M. Shaw, Jane Speight, Pratik Choudhary, Nicole de Zoysa, Stephanie A. Amiel, on behalf of the HypoCOMPaSS Study Group

**Affiliations:** 1 Diabetes Research Group, King’s College London, London, UK; 2 Centre for Psychology at Universidade do Porto, Faculty of Psychology and Educational Sciences, Universidade do Porto, Porto, Portugal; 3 King’s College Hospital NHS Foundation Trust, London, UK; 4 Faculty of Nutrition and Food Sciences, Universidade do Porto, Porto, Portugal; 5 Department of Endocrinology, Diabetes and Metabolism, Centro Hospitalar São João, Porto, Portugal; 6 Faculty of Medicine, Instituto de Investigação e Inovação em Saúde, Universidade do Porto, Porto, Portugal; 7 Translational and Clinical Research Institute, Newcastle University, Newcastle upon Tyne, UK; 8 School of Psychology, Deakin University, Geelong, VIC Australia; 9 The Australian Centre for Behavioural Research in Diabetes, Diabetes Victoria, Melbourne, VIC Australia; 10 Leicester Diabetes Centre, University of Leicester, Leicester, UK

**Keywords:** Hypoglycaemia, Impaired awareness of hypoglycaemia, Type 1 diabetes

## Abstract

**Aims/hypothesis:**

The aims of this study were to assess cognitions relating to hypoglycaemia in adults with type 1 diabetes and impaired awareness of hypoglycaemia before and after the multimodal HypoCOMPaSS intervention, and to determine cognitive predictors of incomplete response (one or more severe hypoglycaemic episodes over 24 months).

**Methods:**

This analysis included 91 adults with type 1 diabetes and impaired awareness of hypoglycaemia who completed the Attitudes to Awareness of Hypoglycaemia (A2A) questionnaire before, 24 weeks and 24 months after the intervention, which comprised a short psycho-educational programme with optimisation of insulin therapy and glucose monitoring.

**Results:**

The age and diabetes duration of the participants were 48±12 and 29±12 years, respectively (mean±SD). At baseline, 91% reported one or more severe hypoglycaemic episodes over the preceding 12 months; this decreased to <20% at 24 weeks and after 24 months (*p*=0.001). The attitudinal barrier ‘hyperglycaemia avoidance prioritised’ (η^2^_p_=0.250, *p*=0.001) decreased from baseline to 24 weeks, and this decrease was maintained at 24 months (mean±SD=5.3±0.3 vs 4.3±0.3 vs 4.0±0.3). The decrease in ‘asymptomatic hypoglycaemia normalised’ from baseline (η^2^_p_=0.113, *p*=0.045) was significant at 24 weeks (1.5±0.3 vs 0.8±0.2). Predictors of incomplete hypoglycaemia response (one or more further episodes of severe hypoglycaemia) were higher baseline rates of severe hypoglycaemia, higher baseline scores for ‘asymptomatic hypoglycaemia normalised’, reduced change in ‘asymptomatic hypoglycaemia normalised’ scores at 24 weeks, and lower baseline ‘hypoglycaemia concern minimised’ scores (all *p*<0.05).

**Conclusions/interpretation:**

Participation in the HypoCOMPaSS RCT was associated with improvements in hypoglycaemia-associated cognitions, with ‘hyperglycaemia avoidance prioritised’ most prevalent. Incomplete prevention of subsequent severe hypoglycaemia episodes was associated with persistence of the cognition ‘asymptomatic hypoglycaemia normalised’. Understanding and addressing cognitive barriers to hypoglycaemia avoidance is important in individuals prone to severe hypoglycaemia episodes.

**Clinical trials registration:**

www.isrctn.org: ISRCTN52164803 and https://eudract.ema.europa.eu: EudraCT2009-015396-27.

**Graphical abstract:**

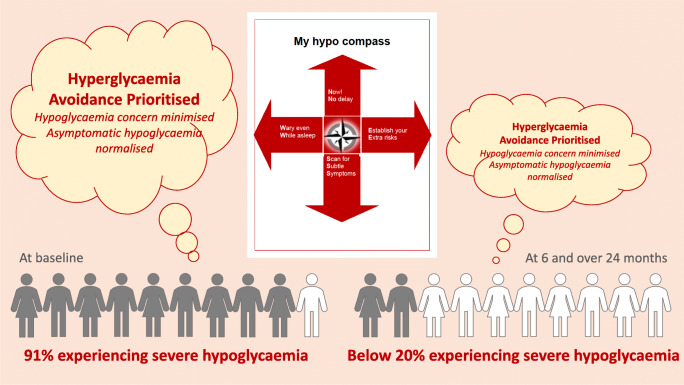

**Supplementary Information:**

The online version contains peer-reviewed but unedited supplementary material available at 10.1007/s00125-022-05847-7.

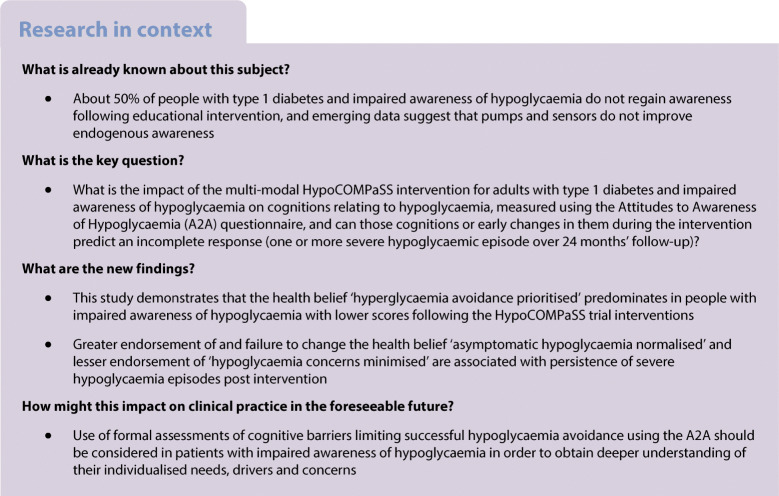



## Introduction

Hypoglycaemia remains a common side effect of the insulin treatment required by people with type 1 diabetes [1, 2], and has not been eliminated through modern diabetes technologies [3, 4]. Hypoglycaemia can be a life-threatening emergency, associated with seizures, coma, cardiovascular events and death [5, 6]. It may lead to fear of hypoglycaemia, with negative impacts on psychological well-being and quality of life [7, 8]. Repeated exposure leads to deficits in normal counter-regulatory responses [9] and impaired awareness of hypoglycaemia (IAH) [10], defined as a diminished ability to perceive the onset of hypoglycaemic symptoms [11]. IAH is associated with a three- to sixfold increased risk of severe hypoglycaemia episodes in patients with type 1 diabetes [4, 11–13]. It affects 20–40% of individuals [1, 4, 12–14], and has been shown to be associated with reluctance to adjust treatment regimens to avoid hypoglycaemia [15]. Severe hypoglycaemia requires third-party assistance for recovery [16] and increases healthcare costs [17–19]. In adults with type 1 diabetes, severe hypoglycaemia has an annual prevalence of about 30% [19], and an annual incidence of 1.3 episodes per person-year [1].

Structured education in flexible insulin therapy and use of diabetes technologies (insulin pumps and glucose sensors) may reduce severe hypoglycaemia episodes, and education may improve IAH without increasing HbA_1c_ [12, 19–24]. These interventions may work by reducing exposure to non-severe episodes, as rigorous avoidance of hypoglycaemia is associated with restored awareness [25, 26]. However, about 50% of people with type 1 diabetes and IAH do not regain awareness following educational intervention [12]. Emerging data suggest that pumps and sensors do not improve endogenous awareness [4, 23, 24]. Qualitative studies suggest that cognitive, behavioural and emotional barriers may prevent people with IAH responding as expected, even to recognised hypoglycaemia [27, 28]. Addressing these barriers may improve outcomes [29]. Attitudinal barriers that may inhibit individuals from taking steps to avoid hypoglycaemia and regain awareness have been assessed using the 19-item Attitudes to Awareness of Hypoglycaemia (A2A) questionnaire, which defines three barriers: ‘asymptomatic hypoglycaemia normalised’, ‘hyperglycaemia avoidance prioritised’ and ‘hypoglycaemia concern minimised’ [30]. The questionnaire was developed based on a qualitative study by Rogers et al [27] in a secondary/tertiary care diabetes clinic in the UK, and was validated for use in a US population of adults with type 1 diabetes [30].

In the HypoCOMPaSS RCT, participants with type 1 diabetes and IAH were allocated to insulin pump therapy (continuous subcutaneous insulin infusion, CSII) or multiple daily insulin injections (MDI) for insulin delivery. All participants optimised glucose monitoring using finger-prick self-monitoring of blood glucose (SMBG) alone or with added real-time continuous glucose monitoring (RT-CGM) [21]. Prior to randomisation, all 96 participants received the ‘my hypo compass’ structured psycho-educational intervention in small groups or individually [31]. The goal was to encourage reflection on personalised factors associated with dangerous hypoglycaemia, leading to formulation of individualised plans to prevent further significant events without increasing exposure to high glucose levels. Participants also received enhanced support from healthcare professionals throughout the 24-week RCT, after which they could switch insulin delivery modality. Those randomised to RT-CGM had uninterrupted continued sensor access. RCT participation was associated with improved hypoglycaemia awareness and a 90% reduction in the severe hypoglycaemia rate at 24 weeks [21], which was maintained at 24 months in parallel with a reduction in HbA_1c_ of 5 mmol/mol (0.5%) [32, 33].

The aims of the present study were to: (1) assess the three A2A attitudinal barriers before and at 24 weeks and 24 months after HypoCOMPaSS RCT participation; and (2) investigate whether A2A attitudinal barriers at baseline, and/or changes from baseline to 24 weeks, were associated with an incomplete response to the intervention, defined as reporting at least one further severe hypoglycaemic episode in the 24-month follow-up [33].

## Methods

Details have been published describing the HypoCOMPaSS study design [31], 24-week RCT outcomes [21] and 24-month follow-up outcomes [32]. In brief, HypoCOMPaSS was an interventional, multicentre, 24-week RCT, with a 2 × 2 factorial design, comparing CSII with MDI and RT-CGM with conventional finger-prick SMBG, with further data collection 24 months after randomisation. Eligible participants were aged 18–74 years with C-peptide-negative type 1 diabetes and IAH, defined by a Gold score ≥4 [11]. All participants attended the psycho-educational intervention ‘my hypo compass’, described below, before being randomised to either CSII or MDI for insulin delivery and RT-CGM or SMBG for glucose monitoring, creating four technology interventions (CSII + RT-CGM, CSII + SMBG, MDI + RT-CGM and MDI + SMBG). After the 24-week data collection period, participants could switch insulin delivery modality (from MDI to CSII or vice versa), whereas the randomised comparison of RT-CGM vs SMBG continued to 24 months, with only those allocated to RT-CGM having access to it. The present analysis was performed across the whole cohort.

During the initial 24-week RCT, everyone received equivalent support/attention from healthcare professionals and attention to therapeutic targets, regardless of the randomised intervention, including four-weekly visits and review of the ‘my hypo compass’ principles, returning to usual care at 24 weeks. The ‘my hypo compass’ programme is a two-hour standardised psycho-educational programme targeting hypoglycaemia avoidance around the four compass points NESW: ‘Now; No delay’ (never delay hypoglycaemia treatment); ‘Establish your Extra risks’ (and times when risk is highest); ‘Scan for Subtle Symptoms’ (of hypoglycaemia); be Wary even While asleep (through watchful detection and active prevention of hypoglycaemia while asleep). Participants completed the A2A questionnaire at baseline, 24 weeks and 24 months [21].

For the present study, the baseline analysis included all participants with complete A2A data prior to randomisation (*n*=91). Those who also had complete A2A data at 24 weeks and 24 months (*n*=54) were included in the analysis of the impact of HypoCOMPaSS on A2A attitudinal barriers over time. To study the relationships between the three A2A attitudinal barriers at baseline or their change over 24 weeks and incomplete response to the intervention (defined by at least one severe hypoglycaemia episode over the 24 months’ follow-up in participants reporting one or more severe hypoglycaemia episode over the 12 months prior to randomisation), 60 participants were included (22 reporting at least one severe hypoglycaemic episode over the 24 month follow-up, and 38 with complete data confirming no severe hypoglycaemia during follow-up). Comparisons of ‘incomplete’ responses vs ‘complete’ responses in all participants were planned before study commencement, with formalisation of definitions preceding any data analysis [33]. As IAH is a risk factor for severe hypoglycaemia but not a guarantee that it will occur, participants without episodes at baseline were not considered appropriate for this pre-specified analysis.

The protocol for the study was approved by Sunderland Research Ethics Committee (09/H0904/63) and the Medicines and Healthcare Products Regulatory Agency (17136/0246/001-0001). The trial was registered (ISRCTN52164803 Eudract number 2009-015396-27). Oversight was provided by an independently chaired Trial Steering Committee and a Data Monitoring and Ethics Committee. Participants gave written informed consent.

### Measures

Episodes of severe hypoglycaemia over the 12 months prior to randomisation were reported, and recorded within the groupings 0; 1; 2; 3 or 4; 5–10; 11–15; 16–20; 21–30; 31–50; >50. Following intervention, episodes were recorded prospectively by participants, and the data were collected by researchers every 24 weeks and expressed within the same frequency groupings as at baseline. To report, annualised rates of severe hypoglycaemia episodes (episodes/person-year) at each time point, means for each grouping were calculated (i.e. 0=0; 1=1; 2=2; 3 or 4=3.5; 5–10=7.5; 11–15=13; 16–20=18; 21–30=25.5; 31–50=40.5; >50=50), and the total number during the 24-week period was multiplied by two for each participant.

IAH was assessed using the Gold score [11]. This asks ‘Do you know when your hypos are commencing?’, with responses rated on a seven-point Likert scale where 1=always aware and 7=never aware. A score ≥4 indicates IAH.

Part 1 of the 19-item A2A questionnaire [30] asks participants to rate their perceived ability, concern and motivation to restore hypoglycaemia awareness on five-point Likert scales where 0=not at all and 4=extremely. Part 2 includes 12 attitudinal statements regarding hypoglycaemia and its avoidance (items numbered within the questionnaire 6–8, 10–12 and 14–19), with responses each ranked on four-point Likert scales where 0=not at all true and 3=very true. They form three factors representing attitudinal barriers to hypoglycaemia avoidance. Items 9 and 13 assess the individual’s perception of their own risk and were not included in this analysis [30]. The factor ‘asymptomatic hypoglycaemia normalised’ (sum of items 6, 7, 10 and 15) assesses the degree to which an individual is motivated to ‘soldier on’ when they have hypoglycaemia (e.g. ‘I don’t need to treat a hypo [low blood glucose] unless I feel symptoms’ and ‘There are no serious consequences to leaving mild hypos [hypoglycemia] untreated’) (US text in square brackets). The factor ‘hyperglycaemia avoidance prioritised’ (sum of items 8, 12, 16 and 19) assesses the level of importance and emotional salience given to avoiding hyperglycaemia over hypoglycaemia (e.g. ‘Good diabetes control is mainly about avoiding high blood glucose levels’ and ‘I get frustrated and/or worried when I see high blood glucose readings’). The factor ‘hypoglycaemia concern minimised’ (sum of items 11, 14, 17 and 18) assesses the degree to which an individual may underestimate the consequences of hypoglycaemia (e.g. ‘Someone will always be around to sort me out [help me] if I go low [have a low blood glucose episode]’ and ‘I don’t get worried very easily [easily worried] about hypos [hypoglycemia]’). For each factor, scores range from 0 to 12, with higher scores representing greater concordance with that belief. The highest of the three scores was used to define the predominant attitudinal barrier for each participant.

At the end of the study, participants were classified into three groups: (1) complete responders (absence of severe hypoglycaemia episodes at all follow-up time points with full severe hypoglycaemia data during follow-up, and one or more severe hypoglycaemia episodes over the 12 months prior to study recruitment); (2) incomplete responders (occurrence of one or more severe hypoglycaemia episodes during the 2-year follow-up, with or without full severe hypoglycaemia follow-up data, and one or more severe hypoglycaemia episodes at baseline); and (3) indeterminate (three subgroups: those without severe hypoglycaemia episodes over the 12 months prior to recruitment and no documented episodes over the 24-month study period, with or without full severe hypoglycaemia follow-up data; those with severe hypoglycaemia episodes at baseline and none documented over the 24-month study period, but with incomplete follow-up data; and those with severe hypoglycaemia episodes at baseline but without follow-up data at all follow-up time points).

### Statistical analyses

Analyses were performed using IBM SPSS 24 for Windows. Descriptive statistics are presented as mean±SD or absolute numbers (*n*) and frequencies (%). The χ^2^ test (or Fisher’s exact test, when the assumptions of the χ^2^ test were violated) was used to compare categorical variables, and unpaired Student’s *t* tests were used to compare continuous variables between the 54 participants with complete A2A data at 24 weeks and 24 months and those with missing A2A data at 24 weeks and/or 24 months (*n*=37). Repeated-measures ANOVA was used to examine the impact of the HypoCOMPaSS intervention on each of the three attitudinal barriers (*n*=54) and the rate of severe hypoglycaemia (*n*=55) (within the 83 people with one or more severe hypoglycaemic episodes at baseline or within the 64 complete or incomplete responders over the 24-month follow-up period), with a post hoc analysis using Sidak’s correction where indicated. Unpaired *t* tests were used to compare the three A2A factor scores at baseline (*n*=64) and the change in scores from baseline to 24 weeks (*n*=62) between complete and incomplete responders. Effect sizes were measured using Cohen’s *d* for data compared by Student’s *t* test, using 0.2, 0.5 and 0.8 to indicate small, medium and large effects [34], and partial eta squared (η^2^_p_) for ANOVA data, using 0.01, 0.06 and 0.14 for small, medium and large effects [35].

Binary logistic regression was used to predict the likelihood of being an incomplete responder to the intervention. The regression was adjusted for severe hypoglycaemia rate at baseline, and sequentially for each of the three A2A attitudinal barriers at baseline; changes in attitudinal barriers from baseline to 24 weeks; clinical site; participant age and diabetes duration. Relationships were considered significant at *p*<0.05.

## Results

### Participants

Of the 96 participants, 82 (85%) had complete data for part 1 of the A2A questionnaire and 91 (95%) had complete data for part 2 of the A2A questionnaire at baseline. Table 1 shows the baseline characteristics of the latter group. By design, all had IAH (Gold score ≥4) and 91% reported severe hypoglycaemia (one or more episode) over the preceding 12 months. The annualised severe hypoglycaemia rate (mean±SD) over the preceding 12 months was high at 8.7±12.7 episodes/person-year (median [IQR] 3.5 [1.0–7.5]). Participants’ characteristics did not differ across the trial sites or the technology arms to which they were randomised.

**Table 1:** Baseline characteristics of the whole cohort

	All participants (*n* = 91) ^a^	
Parameter	Mean ± SD (*n*)	*n* (%)
Age (years)	48.3 ± 12.1 (89)	–
Sex (% female)	–	57 (64.0)
Duration of diabetes (years)	28.9 ± 12.3 (89)	–
BMI (kg/m^2^)	26.6 ± 4.4 (88)	–
HbA_1c_ (mmol/mol)	67.3 ± 13.2 (89)	–
HbA_1c_ (%)	8.3 ± 1.2 (89)	–
Mean SH annualised rate (episodes/person-year)	8.7 ± 12.7 (91)	–
Groupings of SH annualised rate (episodes/person-year)		
0	–	8 (8.8)
1	–	16 (17.6)
2	–	14 (15.4)
3 or 4	–	18 (19.8)
5–10	–	14 (15.4)
11–15	–	6 (6.6)
16–20	–	2 (2.2)
21–30	–	7 (7.7)
31–50	–	1 (1.1)
>50	–	5 (5.5)
IAH (Gold score ≥4)	–	91 (100)
RCT allocation to insulin regimen and monitoring		
MDI with SMBG	–	20 (22.5)
CSII with SMBG	–	23 (25.8)
MDI with RT-CGM and SMBG	–	25 (28.1)
CSII with RT-CGM and SMBG	–	21 (23.6)
Attitudinal barriers (A2A scales)		
Asymptomatic hypoglycaemia normalised	1.5 ± 1.9 (91)	5 (5.5)
Hyperglycaemia avoidance prioritised	5.4 ± 2.3 (91)	76 (83.5)
Hypoglycaemia concern minimised	2.4 ± 1.9 (91)	5 (5.5)
Hyperglycaemia avoidance prioritised plus asymptomatic hypoglycaemia normalised	–	2 (2.2)
Asymptomatic hypoglycaemia normalised plus hypoglycaemia concern minimised	–	1 (1.1)
Hyperglycaemia avoidance prioritised plus hypoglycaemia concern minimised	–	2 (2.2)
Clinical site		
Bournemouth	–	15 (16.5)
Cambridge	–	21 (23.1)
Newcastle	–	21 (23.1)
Plymouth	–	17 (18.7)
Sheffield	–	17 (18.7)

Values are means ± SD (with number of participants in parentheses) for continuous variables or *n* (%) for categorical variables, for which *N* is 91 except for sex (*n* = 89) and RCT allocation to insulin regimen and monitoring (*n* = 89)

SH, severe hypoglycaemia

Following the study intervention, the severe hypoglycaemia rate was reduced, with the annualised rate remaining significantly lower than baseline at all follow-ups (η^2^_p_=0.342, *p*<0.001 for all pairwise comparisons; Table 2), reflecting previously published analyses [21, 31, 32]. The percentage of participants experiencing any severe hypoglycaemia episodes decreased from 91% over the 12 months before intervention to 19% over the first 24 weeks, 16% from 24 weeks–12 months, 13% from 12–18 months, and 17% from 18–24 months (all *p*<0.001). Of the 64 participants for whom data were available, 40 (62.5%) had a complete response over 24 months, with the remaining 24 (37.5%) being incomplete responders. Participants with complete vs missing A2A data at 24 weeks and 24 months (see electronic supplementary material [ESM] Table 1) differed by study site (χ^2^ [4 *df*, *n*=91]=21.10, *p*<0.001) and response to the intervention over the 2-year follow-up (χ^2^ [1 *df*, *n*=64]=7.44, *p*=0.006). The proportion of participants with missing A2A data at 24 weeks and/or 24 months was higher among incomplete responders (50% vs 15%).

**Table 2:** Attitudinal barriers (A2A scale scores) and severe hypoglycaemia episodes at baseline and over the 24 month follow-up period

Parameter	*n*	Baseline	24 weeks	12 months	18 months	24 months	*F*	*p*	η^2^_p_
Attitudinal barriers (A2A scale scores)									
Asymptomatic hypoglycaemia normalised	54	1.5 (0.3)	0.8 (0.2)	–	–	0.9 (0.2)	3.30	0.045	0.113
Hyperglycaemia avoidance prioritised	54	5.3 (0.3)	4.3 (0.3)	–	–	4.0 (0.3)	8.67	0.001	0.250
Hypoglycaemia concern minimised	54	2.3 (0.2)	2.1 (0.2)	–	–	2.3 (0.2)	0.40	0.672	0.015
SH rates (episodes/person-year)	55	8.5 (1.6)	0.5 (0.2)	1.4 (0.7)	0.7 (0.5)	0.6 (0.2)	6.63	<0.001	0.342

Values for scores are estimated marginal means with SD in parentheses, calculated by repeated-measures ANOVA comparing each of the A2A attitudinal barriers and the rates of severe hypoglycaemia over time. The rate of severe hypoglycaemia at baseline was determined from retrospective data reporting for the 12 months prior to recruitment and from prospectively collected data over each 24-week follow-up period, annualised by multiplying by 2, for the 55 participants with severe hypoglycaemia episodes at baseline and full follow-up data. Post hoc tests using Sidak’s correction confirmed a significant difference in the barriers ‘asymptomatic hypoglycaemia normalised’ from baseline to 24 weeks (*p*<0.05) and ‘hyperglycaemia avoidance prioritised’ from baseline to 24 weeks (*p*=0.001) and from baseline to 24 months (*p*=0.004) and in rates of severe hypoglycaemia episodes comparing baseline with other time periods (*p*<0.001 for each). Attitudinal barrier data were not collected at 12 and 18 months

SH, severe hypoglycaemia

### Attitudinal barriers to avoiding hypoglycaemia

In part 1 of the A2A questionnaire, concern about hypoglycaemia was high (3.1±0.9; maximum possible score is 4), with 74% responding ‘a lot’ or ‘extremely’ to the question; likewise motivation to regain awareness was high (3.2±0.8), with 83% responding ‘a lot’ or ‘extremely’, while belief in their ability to regain awareness was lower (1.8±1.0), with 71% responding ‘somewhat’ or ‘slightly’. In part 2 of the questionnaire, the highest concordance rates were for item 8 (‘good’ diabetes management consists mostly in avoiding hyperglycaemia) and item 16 (having hyperglycaemia necessarily causes high levels of frustration and/or anxiety), with 30% and 42%, respectively, responding ‘very true’ (ESM Table 2).

In 84% of participants, the predominant attitudinal barrier to hypoglycaemia avoidance at baseline was ‘hyperglycaemia avoidance prioritised’; in 5.5% ‘asymptomatic hypoglycaemia normalised’; in 5.5%, ‘hypoglycaemia concern minimised’; with the remaining five participants having equal scores on two barriers (Table 1 and ESM Table 1).

Table 2 presents the results for evolution of the attitudinal barriers over time. Compared with baseline, there was a significant reduction (mean=5.3 vs 4.3 vs 4.0, η^2^_p_=0.250) in ‘hyperglycaemia avoidance prioritised’ at 24 weeks (*p*=0.001) and 24 months (*p*=0.004). There was also a significant reduction (η^2^_p_=0.113) in ‘asymptomatic hypoglycaemia normalised’ at 24 weeks (*p*=0.039), which appeared sustained but was no longer statistically significant at 24 months (mean=1.5 vs 0.8 vs 0.9, *p*=0.069). There was no significant change in ‘hypoglycaemia concern minimised’ (mean=2.3 vs 2.1 vs 2.3, η^2^_p_=0.015, *p*=0.672).

### Relationships between attitudinal barriers and incomplete response

Participants with a complete or incomplete response to the HypoCOMPaSS intervention did not differ in baseline values for any attitudinal barrier or the change in those values from baseline to 24 weeks (ESM Table 3).

Table 3 shows the results of binary logistic regression models used to predict likelihood of incomplete response. The models had a good fit to the data (Hosmer–Lemeshow test: *p*>0.05), with Nagelkerke *R*^2^ values of up to 0.445. Higher baseline rates of severe hypoglycaemia episodes predicted an incomplete response to the intervention (*p*<0.05 for all models). Higher baseline values for ‘asymptomatic hypoglycaemia normalised’ (*p*=0.028) and reduced change at 24 weeks (*p*=0.040) also predicted an incomplete response (model B). Conversely, lower baseline values for ‘hypoglycaemia concern minimised’ predicted an incomplete response (*p*<0.05 for models C, D and E). Although participants at Cambridge had a higher probability of an incomplete response compared with the reference site (Sheffield) (*p*<0.05 for models C, D and E), the overall effect of clinical site was not significant (*p*>0.10 for models C, D and E).

**Table 3:** Binary logistic regression models for prediction of incomplete response to the intervention (one or more severe hypoglycaemia episodes over the 24-month follow-up period), adjusting for the three A2A attitudinal barriers at baseline and changes in attitudinal barriers from baseline to 24 weeks, as well as clinical site, participant age, diabetes duration and severe hypoglycaemia rate at baseline

	Model A		Model B		Model C		Model D		Model E	
Parameter	Exp (β)^a^	*p*	Exp (β)^a^	*p*	Exp (β)^a^	*p*	Exp (β)^a^	*p*	Exp (β)^a^	*p*
Baseline score										
Asymptomatic hypoglycaemia normalised	1.15	0.387	2.20	0.028	2.08	0.060	2.14	0.058	2.15	0.061
Hyperglycaemia avoidance prioritised	0.88	0.348	0.89	0.495	0.84	0.327	0.83	0.325	0.83	0.334
Hypoglycaemia concern minimised	0.89	0.526	0.65	0.146	0.45	0.045	0.44	0.045	0.43	0.048
SH rate (episodes/person-years)	1.06	0.013	1.08	0.004	1.13	0.001	1.13	0.001	1.13	0.002
Change from baseline to 24 weeks										
Asymptomatic hypoglycaemia normalised’	–	–	0.51	0.040	0.57	0.104	0.56	0.096	0.56	0.096
Hyperglycaemia avoidance prioritised	–	–	1.01	0.967	0.98	0.924	0.98	0.896	0.98	0.895
Hypoglycaemia concern minimised	–	–	1.18	0.466	1.19	0.469	1.22	0.419	1.23	0.420
Clinical site						0.137		0.144		0.146
Sheffield (reference site)	–	–	–	–	–	(ref.)	–	(ref.)	–	(ref.)
Bournemouth	–	–	–	–	1.00	0.999	1.25	0.854	1.26	0.850
Cambridge	–	–	–	–	28.93	0.014	31.20	0.013	31.40	0.013
Newcastle	–	–	–	–	4.48	0.173	4.54	0.173	4.53	0.174
Plymouth	–	–	–	–	8.84	0.146	9.98	0.131	10.19	0.137
Age	–	–	–	–	–	–	1.02	0.603	1.02	0.628
Diabetes duration	–	–	–	–	–	–	–	–	1.00	0.951
Nagelkerke’s *R*^2^	0.183		0.293		0.441		0.445		0.445	

The numbers of participants included were 38 for a complete response to intervention and 22 for an incomplete response

^a^Values were adjusted to all other variables in each model. Models include: (A) SH rate and A2A attitudinal barriers at baseline; (B) model A with the addition of changes in attitudinal barriers from baseline to 24 weeks (C) model B with the addition of clinical site; (D) model C with the addition of age; (E) model D with the addition of diabetes duration

ref., reference; SH, severe hypoglycaemia

## Discussion

This study measured cognitions relating to hypoglycaemia over a 24-month period in adults with type 1 diabetes and IAH, examining the potential of the HypoCOMPaSS intervention (brief psycho-education and optimisation of insulin treatment with glucose monitoring to reduce hypoglycaemia) to modify attitudinal barriers to hypoglycaemia avoidance, and investigate whether such cognitions, and/or changes in them from baseline to 24 weeks, predict incomplete response to the programme, defined by at least one severe hypoglycaemia episode over the 24-month follow-up period. The predominant attitudinal barrier to hypoglycaemia avoidance in this cohort was ‘hyperglycaemia avoidance prioritised’. This cognition was reduced significantly 24 weeks after the multimodal HypoCOMPaSS intervention, with the change sustained over 24 months. The belief that asymptomatic hypoglycaemia is ‘normal’ was also reduced, being significantly lower after 24 weeks. Stronger endorsement of this cognition at baseline and a reduced change after 24 weeks were associated with an incomplete response during follow-up, as was a lower baseline score for ‘hypoglycaemia concern minimised’.

Participants were recruited to the HypoCOMPaSS RCT due to their well-established problematic hypoglycaemia. At baseline, 74% of participants reported high levels of concern about this, and 83% were highly motivated to regain hypoglycaemia awareness. This contrasts with the qualitative study from which the A2A questionnaire was developed, in which only 24% of people with well-established problematic hypoglycaemia expressed a high level of concern [27]. This is perhaps to be expected, as HypoCOMPaSS participants had volunteered for an intervention study to reduce problematic hypoglycaemia and restore awareness. A Swedish study found that, among the 25% of patients attending their clinic for people with type 1 diabetes who had high risk of severe hypoglycaemia, 68% had appropriately high concern [36], comparable to that for the HypoCOMPaSS cohort. Despite expressing concern regarding significant hypoglycaemia, and motivation to address it, participants showed high endorsement of items in the barrier ‘hyperglycaemia avoidance prioritised’, which may have contributed to their problematic hypoglycaemia risk status. This suggests that concern and motivation alone are not sufficient to prevent problematic hypoglycaemia, and underlying health beliefs (attitudinal barriers) need to be considered.

Endorsement of the barrier ‘hyperglycaemia avoidance prioritised’ fell significantly during the study. A recently published RCT of psycho-educational interventions successfully targeting otherwise treatment-resistant problematic hypoglycaemia in type 1 diabetes also reported changes in cognitions relating to hypoglycaemia [37, 38]. The RCT comparing HARPdoc (Hypoglycaemia Awareness Restoration Programme for adults with type 1 diabetes and problematic hypoglycaemia despite optimised self-care) with blood glucose awareness training showed significant improvements in A2A scores in the HARPdoc arm, with reduction in the ‘hyperglycaemia avoidance prioritised’ barrier (from 6.1 at baseline to 4.0 at 24 months), comparable to that in the current study [38]. That both interventions also showed sustained reductions in severe hypoglycaemia episodes suggests that the magnitude of the change in barrier score to 4.0 is clinically meaningful.

The reduced over-prioritisation of hyperglycaemia after the HypoCOMPaSS intervention detected by the A2A questionnaire was mirrored by significantly reduced ‘worry’ regarding high glucose levels, attenuated ‘low blood glucose’ preference, less ‘avoidance of glucose extremes’, and a lower drive to take ‘immediate action’ for high glucose in participants [33], as reported using the validated Hyperglycaemia Avoidance Scale [39]. Addressing this over-riding drive to ‘avoid high glucose levels at all costs’ is a core tenet of both ‘my hypo compass’ and the intensified support from healthcare professionals over this 24-week RCT. The goal was not to change beliefs that high glucose was ‘dangerous and to be avoided’, but to provide tools targeted at avoidance of hypoglycaemia without an increase in high glucose levels. This was reflected in a mean 8 unit reduction in insulin dose and a reduction of HbA_1c_ at 24 months, with improved hypoglycaemia awareness and reduction of severe hypoglycaemia episodes [32].

The ‘unacceptability’ of any glucose reading <4 mmol/l was emphasised within the ‘my hypo compass’ intervention, which focused on individualised reappraisal of existing knowledge and changing behaviours in light of this reappraisal. Being watchful (W) and acting now (N) without delay were key objectives. In addition, protocol-driven insulin dose reductions were negotiated with participants to address glucose levels under 4 mmol/l. Although the score for ‘asymptomatic hypoglycaemia normalised’ was lower at 24 weeks, the absence of a significant difference from baseline at 24 months suggests that ongoing review of this attitudinal barrier is required in those at continued high risk, with consideration of the need for ‘top-up’ psycho-educational input. The association between further severe hypoglycaemia post-intervention and a higher endorsement of the barrier ‘asymptomatic hypoglycaemia normalised’ at baseline and in participants with the least change in this cognition 24 weeks after intervention provides further support for the need for ongoing monitoring using the A2A questionnaire. The lack of association between continued experience of severe hypoglycaemia episodes and the change in scores for ‘hyperglycaemia avoidance prioritised’ is compatible with the hypothesis that it is lack of change in the less prevalent lower-scoring factor ‘asymptomatic hypoglycaemia normalised’ that is associated with continued experience of severe hypoglycaemia episodes in our cohort. People in this minority group may benefit from more intensive cognitive and psychological support.

‘Hypoglycaemia concern minimised’ was the predominant attitudinal barrier to hypoglycaemia avoidance in only 6% of participants. This was not affected by the HypoCOMPaSS intervention. The association between a lower tendency to minimise these concerns at baseline and ongoing severe hypoglycaemia episodes during the follow-up period is consistent with our previous findings that those continuing to experience severe hypoglycaemia episodes had the highest fear of hypoglycaemia at baseline and at study completion (24 months) [33]. This supports the conclusion that most individuals with the most problematic hypoglycaemia are appropriately concerned regarding the dangers of hypoglycaemia but may be over-accepting of biochemical hypoglycaemia and over-zealous in treating hyperglycaemia.

This study has limitations. As the RCT was designed to compare insulin delivery and glucose self-monitoring modalities, with all participants receiving the psycho-educational intervention and intensified support from healthcare professionals, the impact of ‘my hypo compass’ as a stand-alone intervention has not been determined. Causality cannot be attributed to the described associations, given the lack of a control arm without provision of the ‘my hypo compass’ intervention. However, the highly comparable biomedical and person-reported outcomes between the intervention arms, and the a priori plan to evaluate pre/post outcomes in the whole cohort, alongside an incomplete vs complete responder analysis in all participants, provide at least partial justification for our conclusions. The finding that another psycho-educational intervention targeting cognitions has recently been shown to change A2A scores [38, 40], while its comparator, despite reducing severe hypoglycaemia episodes, did not [38], allows us to speculate that it is the ‘my hypo compass’ component that contributes to the changes seen in the present study. Our findings strongly support inclusion of an educational component when initiating new technologies in individuals experiencing problematic hypoglycaemia, particularly given the absence of evidence for restored hypoglycaemia awareness in trials of technology that lacked a psycho-educational component [23, 24], and the ongoing occurrence of significant hypoglycaemia even on hybrid closed-loop therapy [3]. It appears that such hypoglycaemia is often driven by inappropriate pre-meal and corrective bolusing, potentially associated with over-prioritisation of hyperglycaemia avoidance. A further RCT is planned to determine robustly the impact of ‘my hypo compass’ in comparison with standard care in participants with recurrent severe hypoglycaemia episodes who are commencing hybrid closed-loop therapy.

The current study also has strengths. It specifically recruited adults with type 1 diabetes and IAH. It provided equivalent psycho-education, support/attention from healthcare professionals and therapeutic targets for all participants regardless of randomised technological intervention, with detailed follow-up 18 months after return to standard clinical care. The ‘my hypo compass’ intervention can be delivered by a single trained facilitator in a one-to-one session or a 2-hour group session, with one telephone follow-up/consolidation session 4 weeks later. The multimodal HypoCOMPaSS RCT has demonstrated the feasibility and utility of implementing this in parallel with medical optimisation of conventional self-management supported by diabetes technology. The current study adds to the growing interest in providing tools that help not only to assess hypoglycaemia risk in an individual or group [41] but also characterise that risk with the aim of personalising the intervention pathway, using the A2A questionnaire with its currently unique focus on hypoglycaemia cognitions [42].

In conclusion, this study provides further evidence for the importance of cognitions in hypoglycaemia risk. It shows that ‘hyperglycaemia avoidance prioritised’ is a predominant attitudinal barrier to avoiding hypoglycaemia among some adults with type 1 diabetes, established IAH and severe hypoglycaemia episodes, and that it is possible to address this by a practical intervention. It also suggests that resistance to change of other attitudinal barriers may underpin the failure of such interventions to achieve complete success (i.e. no further severe hypoglycaemia episodes). Formal assessment of the cognitive barriers limiting successful hypoglycaemia avoidance using the A2A questionnaire may enable a deeper understanding of individualised needs, drivers and concerns in those with IAH [42]. This study also provides further evidence for the holistic impact of the HypoCOMPaSS intervention and support for inclusion of the ‘my hypo compass’ intervention as an easy-to-deliver psycho-educational intervention in future trials and clinical programmes focused on sustained avoidance of severe hypoglycaemia episodes.

### Supplementary information

ESM (PDF 181 kb)

## Data Availability

The anonymised datasets generated during and/or analysed during the current study are available from the corresponding authors on reasonable request.

## Appendix

The HypoCOMPaSS Study Group members are S.M. Barendse and J. Speight (previously affiliated with AHP Research, which has ceased trading [2021]); J. Begley, A. Bowes, O. Chapple, D. Kerr and M. Nation (Bournemouth); H. Brown, K. Davenport, M.L. Evans, S. Hartnell, L. Leelarathna, C. Riches and C. Ward (Cambridge); C. Brennand, C. Gordon, A. Lane, S.A. Little, S.M. Marshall, J.A.M. Shaw, J. Stickland, L. Thompson, D.D. Stocken and R. Wood (Newcastle); D. Flanagan, S. Read and H.K. Tan (Plymouth); M. Cunningham, S.R. Heller, S. Hudson, A. Lubina-Solomon, C. Nisbet and E. Walkinshaw (Sheffield). The members of the Trial Steering Committee are S.A. Amiel (chair), J. Begley, C. Brennand, T. Chadwick, E. Davidson, M.L. Evans, D. Flanagan, L. Hall, S.R. Heller, V. King, S. Little, C. Littlewood, J. Matthews, J.A.M. Shaw, C. Speed, J. Speight and R. Wood. The members of the Data Monitoring and Ethics Committee are S. Ashwell, M. Campbell, P.D. Home (chair), D. Kyne and L. Nesbitt.

## Abbreviations

A2A: Attitudes to Awareness of Hypoglycaemia
CSII: Continuous subcutaneous insulin infusion
IAH: Impaired awareness of hypoglycaemia
MDI: Multiple daily insulin injections
RT-CGM: Real-time continuous glucose monitoring
SMBG: Self-monitoring of blood glucose
