# Supplementary material for: Changes in attitudes to awareness of hypoglycaemia during a hypoglycaemia awareness restoration programme are associated with avoidance of further severe hypoglycaemia episodes within 24 months: the A2A in HypoCOMPaSS study
Source: Diabetologia. 2022 Dec 20;66(4):631–41. doi: 10.1007/s00125-022-05847-7 (PMC9947080; doi:10.1007/s00125-022-05847-7)
Supplement: Supplementary file 1 — (PDF 181 kb) [file 125_2022_5847_MOESM1_ESM.pdf]

ESM Table 1 Baseline characteristics of participants in the HypoCOMPaSS randomised controlled trial, comparing the cohort with complete A2A data with those with missing A2A data at 24 weeks and/or 24 months

Baseline characteristics	All participants (n = 91) [a]		Participants with complete A2A data at 24 weeks and 24 months (n = 54)		Participants with missing A2A data at 24 weeks and/or 24 months [b] (n = 37) [c]						
	Mean±SD (n)	n (%)	Mean±SD (n)	n (%)	Mean±SD (n)	n (%)	t	p value	d	χ^2/Fisher	p value
Age (years)	48.3±12.1 (89)	-	50.1±11.8 (54)	-	45.6±12.4 (35)	-	1.71	0.090	0.372	-	-
Sex: Female	-	57 (64.0)	-	35 (64.8)	-	22 (62.9)	-	-		0.00	1.000
Duration of diabetes (years)	28.9±12.3 (89)	-	30.4±13.1 (54)	-	26.5±10.7 (35)	-	1.43	0.156	0.311	-	-
Body mass index (kg/m^2)	26.6±4.4 (88)	-	26.0±4.2 (54)	-	27.4±4.7 (34)	-	-1.46	0.147	-0.320	-	-
HbA1c (mmol/mol)	67.3±13.2 (89)	-	67.1±14.2 (54)	-	67.5±11.6 (35)	-	-0.15	0.882	-0.032	-	-
HbA1c (%)	8.3±1.2 (89)	-	8.3±1.3 (54)	-	8.3±1.1 (35)	-	-0.15	0.880	-0.033	-	-
SH annualised rate (episodes/person-year)	8.7±12.7 (91)	-	8.4±13.6 (54)	-	9.2±11.4 (37)	-	-0.31	0.761	-0.065	-	-
SH annualised rate (episodes/person-year)											
0	-	8 (8.8)	-	-	-	-	-	-			
1	-	16 (17.6)	-	-	-	-	-	-			
2	-	14 (15.4)	-	-	-	-	-	-			
3-4	-	18 (19.8)	-	-	-	-	-	-			
5-10	-	14 (15.4)	-	-	-	-	-	-			
11-15	-	6 (6.6)	-	-	-	-	-	-		-	-
16-20	-	2 (2.2)	-	-	-	-	-	-			
21-30	-	7 (7.7)	-	-	-	-	-	-			
31-50	-	1 (1.1)	-	-	-	-	-	-			
> 50	-	5 (5.5)	-	-	-	-	-	-			

Impaired awareness of hypoglycaemia (Gold score ≥ 4)	-	91 (100)	-	-	-	-	-	-	-	-
RCT allocation to insulin regimen and monitoring										
MDI with SMBG	-	20 (22.5)	-	12 (22.2)	-	8 (22.9)	-	-		
CSII with SMBG	-	23 (25.8)	-	15 (27.8)	-	8 (22.9)	-	-	1.31	0.730
MDI with RT-CGM and SMBG	-	25 (28.1)	-	13 (24.1)	-	12 (34.3)	-	-		
CSII with RT-CGM and SMBG	-	21 (23.6)	-	14 (25.9)	-	7 (20.0)	-	-		
Attitudinal barriers (A2A scales)										
‘Asymptomatic Hypoglycaemia Normalised’	1.5±1.9 (91)	5 (5.5)	1.5±1.9 (54)	4 (7.4)	1.5±1.9 (37)	1 (2.7)	-0.13	0.901	-0.027	
‘Hyperglycaemia Avoidance Prioritised’	5.4±2.3 (91)	76 (83.5)	5.3±2.3 (54)	44 (81.5)	5.7±2.2 (37)	32 (86.5)	-0.92	0.362	-0.195	
‘Hypoglycaemia Concern Minimised’	2.4±1.9 (91)	5 (5.5)	2.3±1.8 (54)	3 (5.6)	2.5±2.1 (37)	2 (5.4)	-0.56	0.580	-0.118	
‘Hyperglycaemia Avoidance Prioritised’/ ‘Asymptomatic Hypoglycaemia Normalised’	-	2 (2.2)	-	2 (3.7)	-	0 (0.0)	-	-	3.62	0.731
‘Asymptomatic Hypoglycaemia Normalised’/ ‘Hypoglycaemia Concern Minimised’	-	1 (1.1)	-	0 (0.0)	-	1 (2.7)	-	-		
‘Hyperglycaemia Avoidance Prioritised’/ ‘Hypoglycaemia Concern Minimised’	-	2 (2.2)	-	1 (1.9)	-	1 (2.7)	-	-		
Clinical site										
Bournemouth	-	15 (16.5)	-	9 (16.7)	-	6 (16.2)	-	-		
Cambridge	-	21 (23.1)	-	16 (29.6)	-	5 (13.5)	-	-		
Newcastle	-	21 (23.1)	-	16 (29.6)	-	5 (13.5)	-	-	21.10	< 0.001
Plymouth	-	17 (18.7)	-	2 (3.7)	-	15 (40.5)	-	-		
Sheffield	-	17 (18.7)	-	11 (20.4)	-	6 (16.2)	-	-		

A2A, Attitudes to Awareness of Hypoglycaemia; CSII, continuous subcutaneous insulin infusion; MDI, multiple daily insulin injections; RCT, randomised controlled trial; RT-CGM, real-time continuous glucose monitoring; SH, severe hypoglycaemia; SMBG, self-monitoring of blood glucose.

Independent-samples *t*-test or Chi-square test (or Fisher's exact test, when chi-square's test assumptions were violated) comparing baseline characteristics between participants with complete A2A data at 24 weeks and 24 months (n = 54) and those with missing A2A data at 24 weeks and/or 24 months (n = 37).

[a] Except for sex (n = 89) and RCT allocation to insulin regimen and monitoring (n = 89). [b]At least one item missing. [c] Except for sex (n = 35) and RCT allocation to insulin regimen and monitoring (n = 35).

ESM Table 2 Description of the A2A items in terms of frequency of participants that fell into each category of response

	All participants (*n* = 91)			
	Categories of response			
	"Not at all true"	*"Slightly true"*	*"Moderately true"*	*"Very true"*
	n (%)			
A2A items				
6. "I don't need to treat a hypo (*US version: low blood glucose*), unless I feel symptoms"	67 (73.6)	12 (13.2)	8 (8.8)	4 (4.4)
7. "I'd rather die living (*live*) life to the full (*fullest*), than be too cautious about my diabetes"	57 (62.4)	24 (26.4)	10 (11.0)	0 (0.0)
8. "Good diabetes control is mainly about avoiding high blood glucose levels"	13 (14.3)	17 (18.7)	34 (37.4)	27 (29.7)
10. "There are no serious consequences to leaving mild hypos (*hypoglycemia*) untreated"	67 (73.6)	20 (22.0)	3 (3.3)	1 (1.1)
11. "Someone will always be around to sort me out (*help me*), if I go low (*have a low blood glucose episode*)"	71 (78.0)	11 (12.1)	7 (7.7)	2 (2.2)
12. "It's more important to avoid going high (*having high blood glucose*) than going low"	39 (42.9)	22 (24.2)	21 (23.1)	9 (9.9)
14. "I can function ok with low (below 3) (*okay with low (<55 mg/dL)*) blood sugar (*glucose*) levels"	28 (30.8)	35 (38.5)	18 (19.8)	10 (11.0)
15. "Treating hypos (*hypoglycemia*) when I don't have symptoms is an unnecessary fuss (*a bother to me*)"	77 (84.6)	8 (8.8)	4 (4.4)	2 (2.2)
16. "I get frustrated and/or worried when I see high blood glucose readings"	5 (5.5)	23 (25.3)	25 (27.5)	38 (41.8)
17. "I don't believe I'll have a severe hypo (*an SH episode*) in the future"	80 (87.9)	7 (7.7)	3 (3.3)	1 (1.1)
18. "I don't get worried very easily (*easily worried*) about hypos (*hypoglycemia*)"	48 (52.7)	26 (28.6)	11 (12.1)	6 (6.6)
19. "Sometimes I know that I am giving myself more insulin than I really need"	55 (60.4)	25 (27.5)	7 (7.7)	4 (4.4)

A2A, Attitudes to Awareness of Hypoglycaemia.

ESM Table 3 Baseline scores and change in scores from baseline to 24 weeks after the HypoCOMPaSS intervention in each of the three A2A attitudinal barriers by intervention response

	Intervention response						
	Complete response: 0 SH episodes from baseline to 24 months		Incomplete response: ≥ 1 SH episode from baseline to 24 months				
	n	Mean (SD)	*n*	Mean (SD)	*t*	*p* value	*d*
Baseline A2A scores							
‘Asymptomatic Hypoglycaemia Normalised’	40	1.5 (1.9)	24	1.8 (2.1)	-0.59	0.555	-0.153
‘Hyperglycaemia Avoidance Prioritised’	40	5.5 (2.6)	24	5.2 (1.7)	0.54	0.592	0.126
‘Hypoglycaemia Concern Minimised’	40	2.4 (1.8)	24	2.6 (2.2)	-0.49	0.625	-0.127
Changes in A2A scores from baseline to 24 weeks							
‘Asymptomatic Hypoglycaemia Normalised’	39	0.8 (1.8)	23	0.7 (2.6)	0.30	0.763	0.080
‘Hyperglycaemia Avoidance Prioritised’	38	0.9 (1.9)	23	0.9 (2.1)	0.02	0.988	0.004
‘Hypoglycaemia Concern Minimised’	39	0.3 (2.3)	23	0.6 (1.3)	-0.58	0.565	-0.152

A2A, Attitudes to Awareness of Hypoglycaemia; SH, severe hypoglycaemia.

T-test comparing baseline A2A scores and change scores from baseline to six months across the two groups.
